# An InDel-based linkage map of hot pepper (*Capsicum annuum*)

**DOI:** 10.1007/s11032-015-0219-3

**Published:** 2015-01-21

**Authors:** Weipeng Li, Jiaowen Cheng, Zhiming Wu, Cheng Qin, Shu Tan, Xin Tang, Junjie Cui, Li Zhang, Kailin Hu

**Affiliations:** 1 College of Horticulture, South China Agricultural University, Wushan Road 483, Guangzhou, 510642 Guangdong China; 2 College of Horticulture and Landscape Architecture, Zhongkai University of Agriculture and Engineering, Zhongkai Road 501, Guangzhou, 510225 Guangdong China; 3 Pepper Institute, Zunyi Academy of Agricultural Sciences, Zunyi, 563102 Guizhou China; 4 Maize Research Institute of Sichuan Agricultural University/Key Laboratory of Biology and Genetic Improvement of Maize in Southwest Region, Ministry of Agriculture, Chengdu, 611130 Sichuan China

**Keywords:** *Capsicum annuum*, InDel, Genetic map, Pepper genome

## Abstract

Two independent pepper (*Capsicum annuum*) genomes were published recently, opening a new era of molecular genetics research on pepper. However, pepper molecular marker technologies are still mainly focusing on the simple sequence repeats derived from public database or genomic library. The development and application of the third generation marker system such as single nucleotide polymorphisms, structure variations as well as insertion/deletion polymorphisms (InDels) is still in its infancy. In the present study, we developed InDel markers for pepper genetic mapping with the convenience of two whole-genome re-sequenced inbred lines BA3 (*C. annuum*) and B702 (*C. annuum*). A total of 154,519 and 149,755 InDel (1–5 bp) sites were identified for BA3 and B702, respectively, by the alignment of re-sequencing reads to Zunla-1 reference genome. Then, 14,498 InDel sites (only 4 and 5 bp) that are different between BA3 and B702 were predicted. Finally, within a random set of 1,000 primer pairs, 251 InDel markers were validated and mapped onto a linkage map using F_2_ population derived from the intraspecific cross BA3 × B702. The first InDel-based map, named as BB-InDel map, consisted of 12 linkage groups, covered a genetic distance of 1,178.01 cM and the average distance between bin markers was 5.01 cM. Compared to the Zunla-1 reference physical map, high consistency was observed on all 12 chromosomes, and the total length of scaffold anchored and physical distance covered by this map was 299.66 and 2,558.68 Mb, respectively, which accounted for 8.95 and 76.38 % of the Zunla-1 reference genome (3.35 Gb), respectively. Furthermore, 37 scaffolds (total length of 36.21 Mb) from the pseudo-chromosome (P0) of the current genome assembly were newly assigned to the corresponding chromosomes by 40 InDel markers. Thus, this map provided good genome coverage and would be useful for basic and applied research in pepper.

**Electronic supplementary material:**

The online version of this article (doi:10.1007/s11032-015-0219-3) contains supplementary material, which is available to authorized users.

## Introduction

The genus of *Capsicum*, which is native to South and Central America (Walsh and Hoot 2001), belongs to the Solanaceae family and includes over 30 species (Moscone et al. 2007). Of these, five are domesticated ones, namely *C. annuum*, *C. chinense* Jacq., *C. baccatum*, *C. pubescens* Ruiz & Pavon and *C. frutescens* (Pickersgill 1997). Due to their characteristic pungency, flavor and nutrient elements, *Capsicum* is cultivated all over the world and becomes one of the most economically important vegetable crops with versatile application for food, spice, ornament, medicine, etc. (Qin et al. 2014). Of the five domesticated species, *C. annuum* is the main cultivated species in China, which is the largest producer and consumer of pepper (www.fao.org). *C. annuum* germplasms have enormous morphological diversity for traits with different fruit size, shape and color (Oyama et al. 2006). Nevertheless, morphological identification can often be problematic when the number of useful traits is limited, which restricts the efficient assessment and utilization of *Capsicum* genetic resources.

Instead, compared to the traditional recognition systems, the DNA marker technology provides a highly reliable tool for rapid and accurate identification of plant species (Jones et al. 2009), which opens a window for us to concern directly on the variations at genomic level, and is now routinely used for study on biodiversity, gene tagging, genetic mapping and marker-assisted selection in various animal and plant systems (Davey et al. 2011; Peleman and van der Voort 2003; Vignal et al. 2002; Sachidanandam et al. 2001). In the last decades, the DNA marker technology of *Capsicum* also experienced the same developmental process of three generations as the other model organisms. Briefly, based on the tomato- and pepper-derived probes, restriction fragment length polymorphism markers (RFLPs) were firstly applied to genetic mapping (Tanksley et al. 1988; Prince et al. 1993) and diversity analysis (Prince et al. 1992; Lefebvre et al. 1993) in *Capsicum.* It was then replaced by PCR-based marker such as amplified fragment length polymorphism (AFLPs) (Paran et al. 1998), random amplified polymorphic DNA (RAPDs) (Rodriguez et al. 1999), simple sequence repeats (SSRs) (Huang et al. 2001; Yi et al. 2006; Lee et al. 2004) and their derived types (Min et al. 2008; Ince et al. 2010; Wu et al. 2009; Du et al. 2006). In recent years, single nucleotide polymorphism markers (SNPs), being known as one of the third generation marker systems, were also started to be used in pepper by different groups (Jung et al. 2010; Jeong et al. 2010; Qin et al. 2014; Kim et al. 2014; Hill et al. 2013).

As one of the most important downstream application of DNA marker, genetic map is also a basic tool necessarily for QTL analysis and marker-assisted selection (MAS) in breeding. In *Capsicum*, genetic maps (Qin et al. 2014; Park et al. 2014; Kim et al. 2014; Sugita et al. 2013; Mimura et al. 2012; Lu et al. 2012b; Wu et al. 2009; Barchi et al. 2007; Yi et al. 2006; Minamiyama et al. 2006; Sugita et al. 2005; Paran et al. 2004; Kang et al. 2001; Livingstone et al. 1999; Tanksley et al. 1988) based on intraspecific or interspecific populations were constructed using various marker systems mentioned above. Even though the SNP brought the density of pepper map to an unprecedented height (Qin et al. 2014; Kim et al. 2014), the total number of PCR-based anchored marker is still limited (Sugita et al. 2013).

Insertion/deletion (InDel) polymorphisms, which were based on sequence alignment, were relatively abundant and uniformly distributed throughout the genome (Mills et al. 2006; Pacurar et al. 2012; Liu et al. 2013). For a species with a reference genome, whole-genome re-sequencing (WDR) can permit the mining of genome data for a large number of genome-wide markers such as SNPs, structure variation (SVs) as well as InDels (Xie et al. 2010; McNally et al. 2009; Qin et al. 2014). With the decreasing cost of next generation sequencing (NGS), the InDels, as a kind of conventional marker to breeder, have been one of the most frequently used markers nowadays (Lv et al. 2013; Liu et al. 2012, 2013; Vasemagi et al. 2010; Ollitrault et al. 2012). So it would be an excellent complement of anchor marker for pepper since the genome sequence was published by two independent groups (Qin et al. 2014; Kim et al. 2014). Nevertheless, to our knowledge, with the exception of a very limited set being identified in silico by comparative transcriptomics (Lu et al. 2011, 2012a), InDel markers have barely been applied to molecular genetics of *Capsicum* practically, such as genetic mapping up to now.

Here, we present the mining of InDels between two *C. annuum* lines BA3 and B702, both of which were already re-sequenced in depth of 28.59- and 30.30-fold, respectively. An InDel-based linkage map of pepper was then constructed using the intraspecific F_2_ population derived from the cross BA3 × B072. The genetic map was then compared with its physical map by anchoring onto the Zunla-1 reference genome. The first InDel map of pepper would be useful for basic and applied research in commercially important cultivated *C. annuum*.

## Materials and methods

### Plant materials and DNA extraction

The F_2_ genetic mapping population consisting of over 300 progenies was derived from the intraspecific cross between two pure lines of *C. annuum* (BA3 × B702) (Qin et al. 2014). In the present study, a random subset of 178 individuals was selected for mapping with InDels. The F_2_ progenies and parental lines were grown in the open field in Zengcheng, Guangzhou City, China. Young leaves were collected for genomic DNA isolation using the CTAB method (Murray and Thompson 1980).

### InDel development and frequency calculation

InDel sites were identified by aligning BA3 and B702 re-sequencing reads to the initial Zunla-1 scaffold genome with SOAPindel (http://soap.genomics.org.cn/), according to the previous study (Qin et al. 2014). To increase the selection efficiency of polymorphic primers, a subset of InDels (only 4 and 5 bp) between BA3 and B702 was predicted by a customized bioinformatic analysis pipeline. Primer3 software (Untergasser et al. 2012) was then used to search primers for each InDel site according to the following parameters settings: (1) predicted product size is between 100 and 300 bp, (2) 5′ and 3′ end mismatch <3 and <1, respectively, and (3) only primers with one hit in the genome assembly were retained. With the accomplishment of chromosome building of reference genome, the retained primers were then anchored onto the final twelve chromosomes (P1–P12) and one pseudo-chromosome (P0) by BLAST (Altschul et al. 1997). InDel frequency was calculated by the formula: (number of heterozygote × 1 + number of homozygote × 2)/2*N*, *N* = total number of accessions.

### PCR amplification and marker scoring

Polymorphic markers that were unique to either of the parental lines and present in the F_1_ population were used for genetic mapping. PCR mixture contained 10 ng genomic DNA, 100 μM of each dNTP, 1.5 μM of each primer, 1× reaction buffer (including Mg^2+^) and 0.5 unit of *Taq* polymerase (Dsbio) in a final volume of 20 μL. The reaction was performed as follows: an initial 5 min at 94 °C; 35 cycles of 45 s at 94 °C, 45 s at 55–58 °C, and 2 min at 72 °C, and a final 10 min at 72 °C. Subsequently, 2–4 μL of the PCR product was used for electrophoresis in 6 % polyacrylamide gel.

### Linkage map construction and comparison with physical map

Linkage analysis was performed using JoinMap 4.0 software (Van Ooijen 2006). Since the physical mapping information on the polymorphic markers was available, groups were firstly created with the command “*Create Groups Using a Map Node*,” the remaining unmapped (actually anchored onto the P0) markers were assigned to the known groups with the *Strongest Cross Link* information. Regression algorithm was used for mapping on each group. Recombination values were converted to genetic distances using the Kosambi mapping function. The genetic map and physical map were drawn using Mapchart 2.2 software (Voorrips 2002). Markers with segregation ratios that differed from expected ratio were classified as segregation distortion markers. A region with five or more adjacent skewed segregation marker was defined as a segregation distortion region (SDR).

## Results and discussion

### Development of the InDel markers

Re-sequencing could help us to discover genome-wide variations on a large scale and provide excellent resources to the plant science community (Albert and Chang 2014). With the ongoing of pepper genome project, we re-sequenced a total of more than 20 different cultivated varieties including the parental lines BA3 and B702 used in the present study (Qin et al. 2014). Based on the alignment of the sequencing reads corresponding to 28.59- (for BA3) and 30.30 (for B702)-fold depth to the reference, 154,519 and 149,755 small InDels (1–5 bp) were identified in BA3 and B702, respectively. Through comparative analysis by a customized bioinformatic analysis pipeline, 14,498 InDels (only 4 and 5 bp) were identified between BA3 and B702 and used for searching primers. Finally, according to the requirements of primer design, a total of 2,324 (16.03 %) primer pairs were successfully obtained. To evaluate their potential value in practice, a random subset of 1,000 primer pairs were chosen to screen polymorphism between BA3 and B702 and 922 (92.2 %) were amplified specifically, indicating the high quality of the reference genome. Two hundred and seventy-two (27.2 %) polymorphic markers were validated, and the majority of the polymorphic markers (>96 %) are codominant inheritance. As expected, the polymorphic rate became lower (13.1 %) when used to test in another pair of parental lines, BA3 and YNXML (*C. frutescens*) in our laboratory (unpublished data).

### Construction of the InDel map

**Fig. 1:**
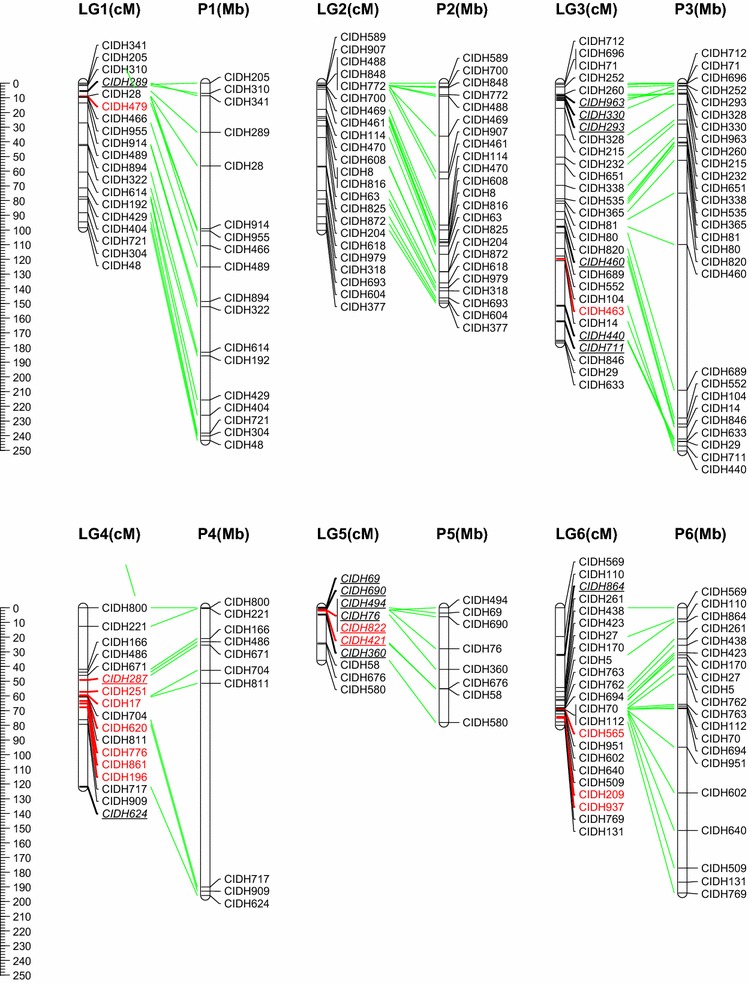

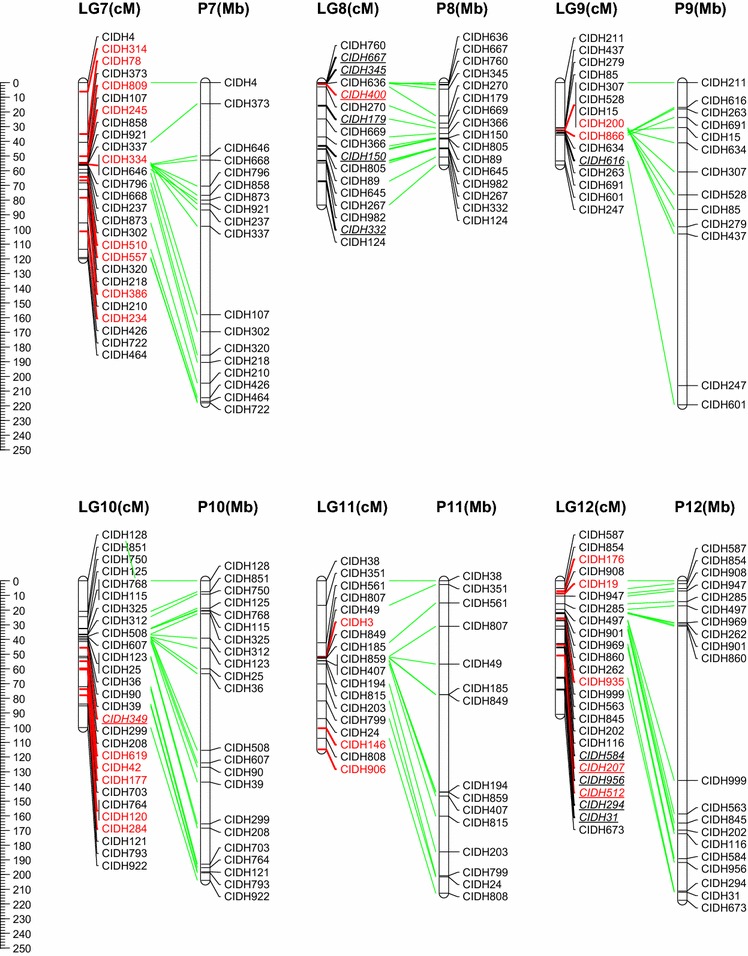
InDel-based linkage map of pepper (*C. annuum*) and comparison with its physical map. All InDel markers start with a prefix ‘CIDH’. Markers written with *red color* are anchored onto the pseudo-chromosome (P0) of the BB-SNP map-based reference genome assembly. A total of 31 distorted segregation markers mapped on this map are underlined and italic. (Color figure online)

**Fig. 2:**
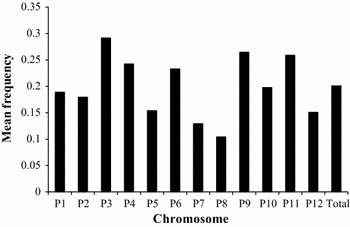
Mean frequency of 251 markers mapped on the BB-InDel map among 17 re-sequenced *C. annuum* accessions

The F_2_ population consisting of 178 progenies derived from the cross BA3 × B702 was genotyped with the above InDel markers with very less missing rate (<1.7 %). An intraspecific linkage map of *C. annuum*, designated as the BB-InDel map, was built with 251 InDel markers (236 genetic bins), and the remaining 21 markers could not be integrated because of insufficient linkage (Fig. 1). This is the first report on the construction of intraspecific linkage map purely based on InDel markers for pepper. In order to evaluate the transferability of InDel markers among *C. annuum* accessions, InDel frequency was calculated using the re-sequencing data from a set of *C. annuum* accessions. The mean frequency of 251 mapped InDels markers among the 17 *C. annuum* accessions was 20.07 % (Fig. 2), indicating that the BB-InDel map can be used for basic and applied research in the future.

The map consisted of 12 linkage groups (LGs) covering a total genetic distance of 1,178.01 cM with an average density of one bin marker for every 5.01 cM (Table 1; Fig. 1). The maximum genetic distance between two bin markers was 42.44 cM, and the number of mapped markers on LGs ranged from 8 (LG5) to 28 (LG3). Segregation distortion (SD) occurs when the segregation ratio deviates from the expected Mendelian ratio (Kuittinen et al. 2004). Here, 35 out of 272 (12.9 %) markers showed distorted segregation, which is lower than that of interspecific population (Kang et al. 2001; Livingstone et al. 1999) but is similar to the intraspecific crossing (Barchi et al. 2007; Sugita et al. 2005; Lefebvre et al. 2002). Two segregation distortion regions (SDRs) were detected on LG5 and LG12, respectively (*P* < 0.05). All marker alleles within the SDR in LG5 were associated with the male line B702, and the other SDR in LG12 were skewed toward the hybrid of the parental lines. The phenomenon might be resulted from some so-called segregation distorted factors (Lyttle 1991), which could alter the recombination frequency in these regions.

**Table 1:** Statistics of the pepper InDel-based linkage map and improvement for current assembly of Zunla-1 genome

LG (chromo some)	Mapped		Bin distance (cM)			Map length			Improvement for assembly		
	Marker	Bin	Average	Min	Max	Genetic (cM)	Anchored (Mb)^a^	Physical (Mb)^b^	Marker^c^	Scaffold	Length (Mb)
LG1 (P1)	19	19	5.47	0.16	18.09	98.41	24.81	288.89	1	1	0.44
LG2 (P2)	23	20	5.28	0.18	19.53	100.24	25.99	162.43	0	0	–
LG3 (P3)	29	28	6.55	0.82	30.76	176.82	28.41	253.84	1	1	0.09
LG4 (P4)	17	17	7.62	0.32	42.44	121.84	23.92	205.50	7	5	8.25
LG5 (P5)	10	8	5.11	0.21	19.43	35.77	13.53	200.29	2	2	3.49
LG6 (P6)	23	22	3.81	0.14	22.17	79.98	30.25	206.18	3	3	3.06
LG7 (P7)	27	26	4.79	0.02	28.88	119.87	32.29	221.90	9	9	7.64
LG8 (P8)	17	17	5.21	0.10	16.19	83.32	17.76	152.79	1	1	0.59
LG9 (P9)	15	12	5.10	0.10	30.70	56.07	14.53	226.90	2	1	2.48
LG10 (P10)	28	25	4.16	0.12	20.88	99.92	40.09	205.30	6	6	8.43
LG11 (P11)	18	17	7.18	0.21	25.42	114.80	21.41	205.17	3	3	0.54
LG12 (P12)	25	25	3.79	0.02	16.98	90.97	26.67	229.49	5	5	1.19
Total	251	236	5.01	–	–	1,178.01	299.66	2,558.68	40	37	36.21

^a^Total length of scaffolds anchored by InDel markers

^b^Spanned length on current Zunla-1 assembly

^c^Number of marker that anchored onto the chromosome P0 in the Zunla-1 reference genome

### Genetic and physical map comparison

Initially, the discovery of InDels was based on the alignment of re-sequencing reads to the Zunla-1 scaffold genome. With the accomplishment of chromosome building of the Zunla-1 reference genome (http://peppersequence.genomics.cn) and the coordinate conversion of scaffold to that of chromosome, the 12 LGs were successfully assigned to the corresponding 12 chromosomes (P1–P12) based on the 211 anchored markers (Table 1 and S1, Fig. 1). The remaining 40 markers are mapped onto the pseudo-chromosome (P0) according to the current assembly of reference genome. They scattered on 37 different scaffolds, spanning a total length of 36.21 Mb (Table 1 and S1). Because the Zunla-1 chromosome building is based on the BB-SNP map (Qin et al. 2014), which is developed using the same F2 population derived from the cross BA3 × B702, the 37 scaffolds would be suggestibly assembled into the corresponding chromosomes (P1–P12) based on this InDel linkage map, providing a reference of genome improvement in some degree.

According to the comparative analysis, we found that the consistency between the genetic and physical position on all 12 chromosomes was high (Fig. 1). The total length of scaffold anchored and physical distance covered by this map is 299.66 and 2,558.68 Mb, respectively (Table 1), which accounted for 8.95 and 76.38 % of the Zunla-1 reference genome (3.35 Gb), respectively. However, there were still some inconsistent orders within certain a very limited region, which was possibly caused by the different mapping algorithm or putative homology-based scaffold orientation (Qin et al. 2014). Nevertheless, clustering of markers around the putative centromeric regions was evidently observed on several chromosomes such as P3, P4, P6, P10–P12 (Fig. 1). On the other hand, plots of genetic versus physical distance also permitted us to observe S shape on all chromosomes with exception of P1, P2, P5 and P8 (Fig. S1). This is seemly normal for P2 and P8 because they are acrocentric chromosomes in *C. annuum* (Lanteri and Pickersgill 1993). In terms of P1 and P5, the relatively low densities of one marker per 10.84 Mb may be the main reason and the plots provided clear reference for the following map saturation. Therefore, these data showed that the BB-InDel map covered nearly the entire genome and could serve as a basic reference map for future genetics and QTL analysis in pepper.

## Conclusions

Re-sequencing technology permitted the mining of over ten thousand small InDels (4 and 5 bp) between two elite inbred lines of *C. annuum*. As a pilot study on the application of recently published pepper genome, 272 polymorphic InDel markers were validated and a genetic map was constructed with 251 purely InDel markers. Comparison between the genetic and physical map indicated the good genome coverage of the map. Therefore, the InDel markers and map present here provided a collection of publicly available anchor markers and will be useful for genetic/QTL analysis in pepper.

## Electronic supplementary material

Below is the link to the electronic supplementary material.

Supplementary material 1 (DOCX 434 kb)

Supplementary material 2 (XLSX 10 kb)

Supplementary material 3 (XLSX 80 kb)
