# Supplementary material for: An InDel-based linkage map of hot pepper (Capsicum annuum)
Source: Mol Breed. 2015 Jan 21;35(1):32. doi: 10.1007/s11032-015-0219-3 (PMC4300394; doi:10.1007/s11032-015-0219-3)
Supplement: Supplementary file 1 — Supplementary material 1 (DOCX 434 kb) [file 11032_2015_219_MOESM1_ESM.docx]

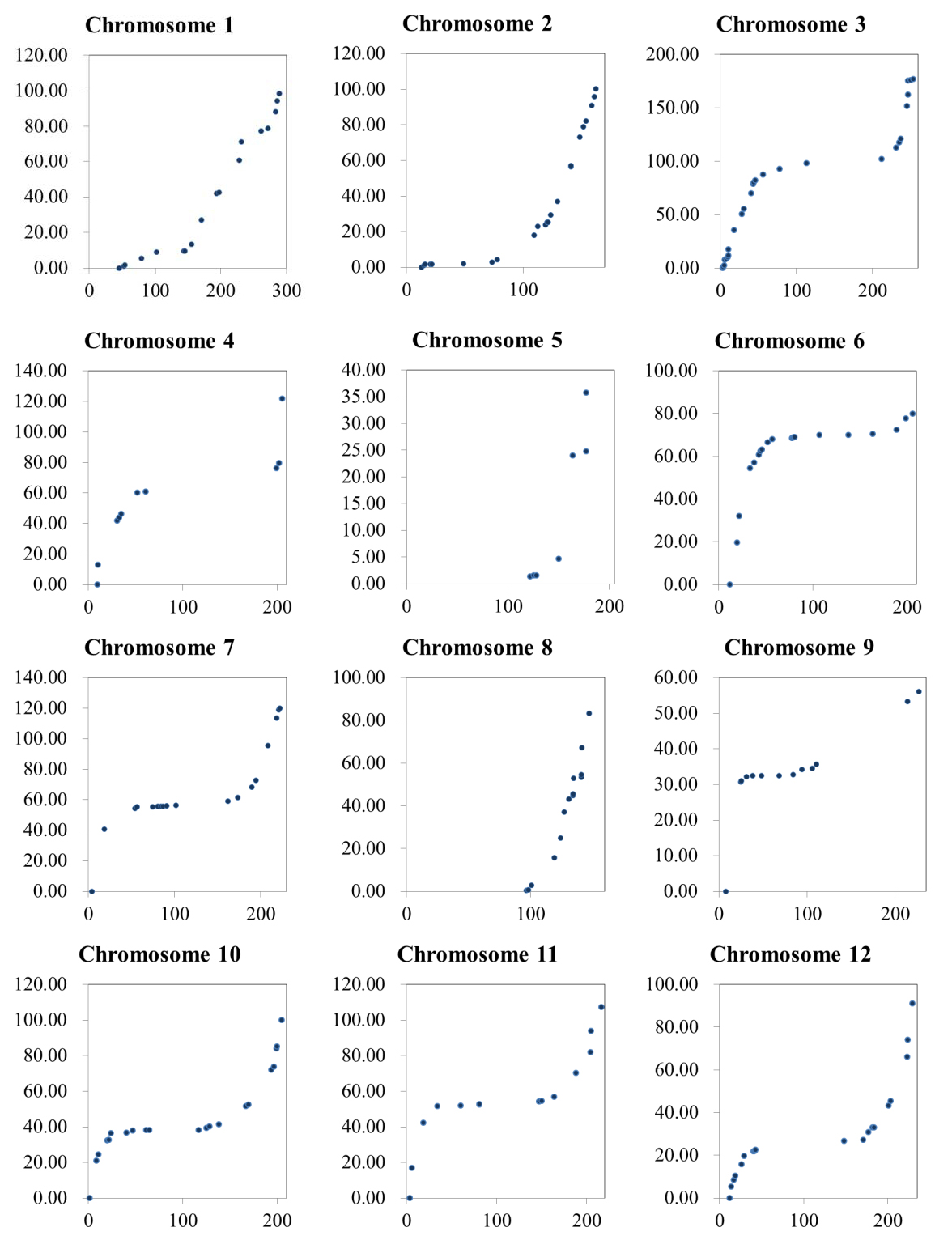


**Fig. S1** Plots of genetic versus physical distance for each chromosome. Horizontal axis: physical distance (in Mb). Vertical axis: geneticdistance (in cM).
